# Antimicrobial, Anticancer, and Antioxidant Activities of Maize and Clover Pollen Grains Extracts: A Comparative Study with Phytochemical Characterizations

**DOI:** 10.3390/ph16121731

**Published:** 2023-12-15

**Authors:** Heba Barnawi, Husam Qanash, Abdu Aldarhami, Ghaida Alsaif, Bandar Alharbi, Majed N. Almashjary, Alhomidi Almotiri, Abdulrahman S. Bazaid

**Affiliations:** 1 Department of Medical Laboratory Science, College of Applied Medical Sciences, University of Ha’il, Hail 55476, Saudi Arabia; h.barnawi@uoh.edu.sa (H.B.); h.qanash@uoh.edu.sa (H.Q.); g.alsaif@uoh.edu.sa (G.A.); b.alharbi@uoh.edu.sa (B.A.); 2 Medical and Diagnostic Research Center, University of Ha’il, Hail 55473, Saudi Arabia; 3 Department of Medical Microbiology, Qunfudah Faculty of Medicine, Umm Al-Qura University, Al-Qunfudah 21961, Saudi Arabia; ahdarhami@uqu.edu.sa; 4 Department of Medical Laboratory Sciences, Faculty of Applied Medical Sciences, King Abdulaziz University, Jeddah 22254, Saudi Arabia; malmashjary@kau.edu.sa; 5 Hematology Research Unit, King Fahd Medical Research Center, King Abdulaziz University, Jeddah 22254, Saudi Arabia; 6 Department of Clinical Laboratory Sciences, College of Applied Medical Sciences-Dawadmi, Shaqra University, Dawadmi 17464, Saudi Arabia; hsalmutiri@su.edu.sa

**Keywords:** biological activities, pollen grains, flavonoids, phenols, cytotoxicity, antibacterial mechanism

## Abstract

The failure to treat infectious diseases due to the continual emergence of drug-resistant microbes poses a huge and serious challenge for human health globally. Currently, the discovery and development of natural therapeutic compounds are attracting considerable attention from researchers worldwide. In this project, two types of pollen grains (maize and clover) were evaluated for potential antimicrobial activities. Extracts of both pollen grains were purified using HPLC, which has been shown to have numerous phenolic and flavonoid compounds. Pyro catechol and methyl gallate were detected in high concentrations (1145.56 and 1056.57 µg/mL, respectively) in the maize extract, while caffeic acid, quercetin, and kaempferol (464.73, 393.05, and 390.93 µg/mL, respectively) were among the compounds observed at high concentrations in the clover pollen grains extract. Staphylococcus aureus, Escherichia coli, Salmonella typhi, and Candida albicans were more sensitive to the clover pollen grains extract with inhibition zones of 22 ± 0.2, 18 ± 0.1, 29 ± 0.3, and 42 ± 0.4 mm compared to the size of the inhibitory zones caused by the maize pollen grains extract (19 ± 0.3, 15 ± 0.4, 27 ± 0.1, and 22 ± 0.4 mm, respectively). Moreover, lower MIC values for the clover pollen grains extract were recorded against C. albicans (1.97 ± 0.04 µg/mL), S. aureus (62.5 ± 1.00 µg/mL), and E. coli (62.5 ± 0.07 µg/mL) than the MICs caused by the maize pollen grains extract. The use of a transmission electron microscope revealed that the E. coli that had been treated with the clover pollen grains extract showed changes in its cell walls compared to that treated with the maize pollen grains extract. The clover pollen grains extract exhibited a stronger antioxidant potential, with an IC50 value of 22.18 µg/mL, compared to an IC50 value of 54.85 µg/mL for the maize pollen grains extract, via a DPPH scavenging assay. Regarding anticancer activity, the maize pollen grains extract was revealed to be more effective in terms of inhibiting the human colon cancer cell line HCT-116, with an IC50 value of 67.02 ± 1.37 µg/mL, compared with the observed toxicity caused by the clover extract, with an IC50 value of 75.03 ± 1.02 µg/mL. Overall, the clover pollen grains extract demonstrated potent antibacterial and antioxidant activities, but not anticancer activity, when compared to the maize grains extract. Thus, the current findings related to both types of pollen grains (clover and maize) highlight their potential therapeutic applications for the treatment of certain infectious diseases and malignancies.

## 1. Introduction

Pollen grains are well known as traditional medicines in certain countries due to their numerous effective phytoconstituents, including steroids carotenoids, flavonoids, terpenoids, and phenolics [1]. Pollen grains’ antimicrobial and antioxidant efficiency, as well as their phenolic composition, have been investigated in several studies; for instance, Avşar et al. [2] mentioned that pollen grains of *Castanea sativa* have displayed antimicrobial activity toward certain Gram-positive and Gram-negative bacteria but were less potent against yeasts, although a strong antioxidant activity was reported. Date palm pollen grains have been shown to exhibit high flavonoid and phenolic contents with promising antibacterial and antioxidant activities [3]. Previously, Almaraz-Abarca et al. [4] reported that the antioxidant potential of pollen grains is mainly due to the presence of flavonoid and phenolic compounds. Moreover, anti-inflammatory, anti-toxicant, and hepato-protective properties have been attributed to pollen grains [5,6,7].

The correlation between the origin of plants’ chemical and biological activities, such as antibacterial and antioxidant activities, is fundamental for further studies [8,9,10]. Velásquez et al. [11] studied the relationship among a plant’s origin, its phytoconstituents, and the observed antibacterial properties of its pollen grains. In addition, the same group of researchers have reported that the pollen grains of *Galega officinalis* L. and *Brassica* species have inhibited the growth of *Escherichia coli*, *Pseudomonas aeruginosa*, *Streptococcus pyogenes*, and *Staphylococcus aureus*, with higher efficacy than conventional antibiotics. The anticancer activity of pollen grains was reported by Kaur et al. [12], but it was linked with a certain type of plant, including *Cassia glauca*, followed by *Bauhinia variegate*, *Cassia siamea,* and *Cassia biflora*. Several phenolic and flavonoid compounds have been detected in plant pollen grains, such as naringenin protocatechuic acid, gallic acid, hesperidin, kaempferol, isorhamnetin 3-orutinoside, quercetin, isorhamnetin, rutin, vanillic acid, quercetin 3-*o*-neohesperidoside, luteolin, 3-*o*-rutinoside, *p*-coumaric acid, apigenin, and rhamnetin 3-*o*-neohesperidoride [13]. Chantarudee et al. [14] reported the presence of 0.567 µg/g biotin, 0.199 µg/g invert sugar, and 0.0153 mg β carotene and vitamin A in the pollen grains of corn. Moniruzzaman et al. [15] have linked the antioxidant activity of pollen grains to its high contents of phenolics and flavonoids. 

Developments in the utilization of drugs for cancer management are often correlated with unfavorable side effects or recurrence. Mărgăoan et al. [16] revealed the application of pollen grains for cancer treatment. One study in the literature claimed that the polyphenols of pollen grains are one of the main reasons behind their anticancer activity and apoptosis stimulation [17]. Pollen grains have shown antibacterial activity toward certain bacteria, including *E. coli*, *Staphylococcus aureus,* and *Bacillus subtilis*, but have not shown antifungal activity [18]. In the same study, anticancer activity was observed against HepG-2 cancer, Caco-2 cells, and PC3 cell lines with different IC_50_ values. Recently, Bakour et al. [19] studied the antimicrobial and antioxidant potential of pollen grains for six different plants, including *Quercus ilex, Punica granatum, Centaurium erythraea, Ruta graveolens, Citrus aurantium*, and *Coriandrum sativum*. Pollen grains of *P. granatum* and *Q. ilex* demonstrated a significant inhibition for the growth of *Pseudomonas aeruginosa*, *E. coli*, *Enterobacter cloacae*, *Klebsiella pneumoniae, Staphylococcus aureus,* and *Acinetobacter baumannii* than *C. sativum, C. erythraea,* and *R. graveolens*, while no antibacterial activity was observed for *C. aurantium*. To the best of our knowledge, the data related to the comparative bioactivities of clover and maize pollen grains, especially those collected prior to honeybees entering their hives, are absolutely absent or very limited. Thus, the main aim of this study was to assess the potential antimicrobial, anticancer, and antioxidant activities of the collected clover and maize pollen grains as well as to provide a characterization of their detected phenolic and flavonoid compounds.

## 2. Results and Discussion

### 2.1. Phytoconstituents Characterizations

**Figure 1:**
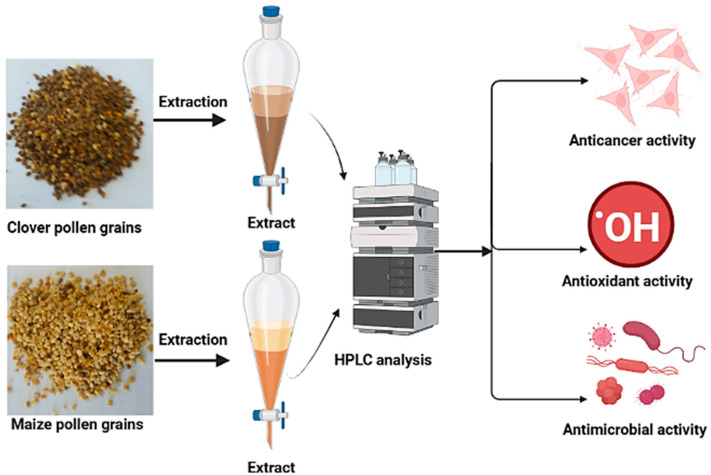
Image summarizing the performed tests for maize and clover pollen grains.

In the present investigation, two types of pollen grains were investigated to evaluate their phenolic and flavonoid contents and their biological activity (Figure 1). An HPLC analysis of the phenolic and flavonoid contents of maize and clover pollen grains was reported (Supplementary Figures S1 and S2) and recorded (Table 1). The pollen grains from the maize pollen grains extract were characterized by the presence of a high amount of pyro catechol (1145.56 µg/mL), followed by methyl gallate (1056.57 µg/mL), and low amount of ferulic acid (5.88 µg/mL), ellagic acid (6.26 µg/mL), and daidzein (7.65 µg/mL). On the other hand, caffeic acid (464.73 µg/mL), quercetin (393.05 µg/mL), and kaempferol 390.93 µg/mL) had the highest detected concentrations, while naringenin (4.07 µg/mL) and vanillin (4.42 µg/mL) had the lowest concentrations in the clover pollen grains. Ten out of the eighteen detected compounds were recorded at higher concentrations in the clover pollen grains than in the maize pollen grains, including catechin, caffeic acid, ellagic acid, ferulic acid, rosmarinic acid, daidzein, quercetin, cinnamic acid, kaempferol, and hesperetin. Seven out of the eighteen detected compounds were recorded at higher concentrations in the maize pollen grains than in the clover pollen grains, except for rutin (68.41 µg/mL), which was in the maize pollen grains only. Therefore, the yellow color of maize pollen grains is due to the presence of rutin and quercetin [20]. In a previous study, 14 types of pollen grains were investigated to detect the flavonoid and phenolic constituents. Caffeic acid derivatives were shown to be the majority of the examined pollen grains [21]. In contrast, there were several phenolic and flavonoid compounds in two types of pollen grains, but their levels depended on the type of pollen grains. Nevertheless, the level/concentration of phenolic and flavonoid compounds in these pollens was dependent on geographical origin and climatic conditions as well as plant status. According to certain scientific papers on the constituents of pollen grains, kaempferol quercetin, naringenin, and caffeic acid represented the main functional constituents in biological activities [22]. A previous study associated with clover and maize pollen grains [23] has reported *p*-coumaric acid, catechin, daidzin, cinnamic acid, and pyrogallic acid as the main constituents of clover pollen grains, followed by caffeic and *p*-hydroxy benzoic acids, gestein and ferulic acid; however, eugenol and kaempferol were present in trace amounts. In addition, pollen grains of maize contain high amounts of cinnamic acid, p-coumaric acid, daidzin, and *p*-hydroxy benzoic, moderate amounts of caffeic acid, ferulic acid and genistein, and trace amounts of eugenol and kaempferol. 

### 2.2. Antimicrobial Activity of Maize and Clover Pollen Grains Extract

**Figure 2:**
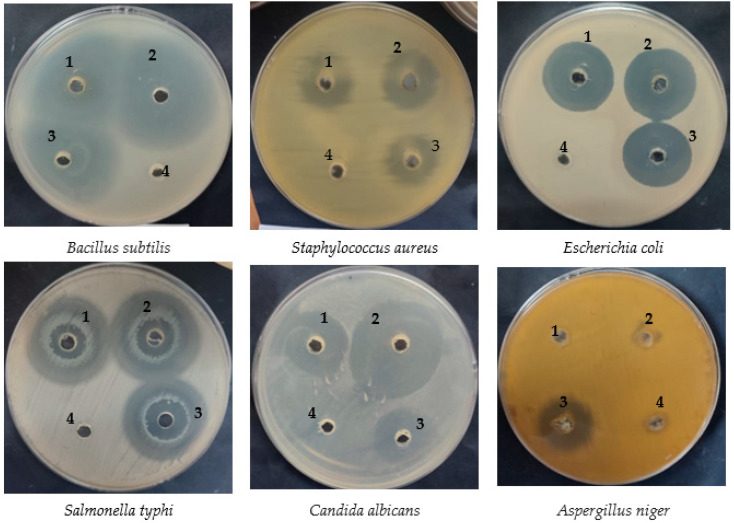
Antimicrobial activity of maize (1) and clover (2) pollen grains extracts compared to the positive (3) and negative (4) controls.

The pollen grains extract of clover exhibited antibacterial activity with more inhibition zones of 22 ± 0.2, 18 ± 0.1, 29 ± 0.3, and 42 ± 0.4 mm, compared to the maize grains extract which showed inhibition zones of 19 ± 0.3, 15 ± 0.4, 27 ± 0.1 and 22 ± 0.4 mm against some tested microorganisms, including *S. aureus*, *E. coli*, *S. typhi*, and *C. albicans*, respectively. However, *B. subtilis* was more sensitive to the maize pollen grains extract (with an inhibition zone of 42 ± 0.4 mm) than the clover pollen grains extract (with an inhibition zone of 39 ± 0.3 mm) (Figure 2 and Table 2). Furthermore, there was no differences between the MIC of the maize and clover pollen grains extract for *B. subtilis* and *S. typhi*, while a low MIC of the clover pollen grains extract was observed against *C. albicans* (1.97 ± 0.04 µg/mL), *S. aureus* (62.5 ± 1.00 µg/mL), and *E. coli* (62.5 ± 0.07 µg/mL) compared to the MIC of maize pollen grains. In addition, the MBC of the clover pollen grains extract was less than the MBC of the maize pollen grains extract against all tested microorganisms except *S. typhi*. The two pollen grains extracts reflected bactericidal properties against all tested microorganisms. This explanation was documented via the estimation of MBC/MIC index (Table 2). The filamentous fungus (*A. niger*) was resistant to both the maize and clover pollen grains extracts. Results of the current study were in agreement with an earlier report, where ethanolic and aqueous extracts of clover pollen grains exhibited higher inhibition zones (26.70 and 25.40 mm, respectively) than ethanolic and aqueous extracts of maize (20.75 and 22.65 mm, respectively) against *Paenibacillus larvae* [23]. However, Khider et al. [24] reported different results, where the highest antibacterial activity was attributed to the methanolic extract of maize pollen grains, which was followed by clover with MIC values of 320–640 μg/mL using clover pollen grains and of 320–1280 μg/mL using maize pollen grains against tested bacteria. Khider et al. collected the pollen grains from inside the honeybee hives. In this study, however, the pollen grains were collected prior to the honeybees entering their hives. In addition to the antibacterial activity of quercetin and kaempferol (contents of pollen grains extract), they showed anti-yeast activity against *Candida parapsilosis* [25]. *Enterococcus faecalis* was inhibited by the pollen grains extract of *Ailanthus altissima*, *Cupressus arizonica* and *Chenopodium album* with different MIC values of 38.8, 113, and 562 µg/mL, respectively [26]. Concerning antibacterial activity, earlier reports indicated that the natural compounds could possess significant antibacterial activity if their MIC was fewer than 100 µg/mL [27].

**Figure 3:**
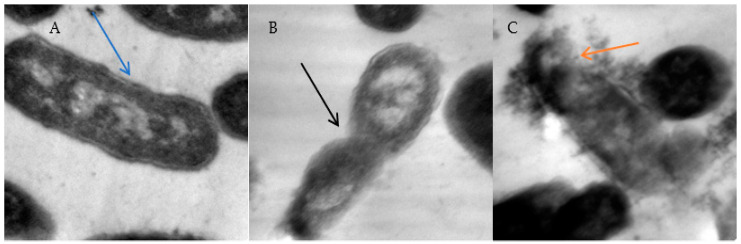
Ultrastructure of *E. coli* (**A**), *E. coli* exposed to maize (**B**) and *E. coli* exposed to clover (**C**) pollen grains extract. Direct magnification 6000×.

The effect of maize and clover pollen grains on the ultrastructure of *E. coli* was examined and imaged (Figure 3). The impact of two types of pollen grains extract was assessed on the cell structure of *E. coli* compared to unaffected *E. coli*. Clover pollen grains were more effective than maize pollen grains. Cell wall deformation was observed in case of the effect of maize pollen grains (black arrow) with a leakage of cytoplasm because of cell wall rupture (orange arrow). In contrast, control cells of *E. coli* were examined in an identical shape with the presence of perfect and clear healthy cell walls, including membranes and cytoplasm (blue arrows).

### 2.3. Antioxidant Activity of Maize and Clover Pollen Grains Extract

Extracts of both pollen grains, including maize and clover exhibited antioxidant activity with an increased DPPH scavenging (%) as their concentration was increased. The current results reflected that the clover pollen grains extract had the highest capacity of antioxidant out of all the maize grains at all tested concentrations. DPPH scavenging (%) was 24.1 and 12.1% at 1.95 µg/mL, 68.1 and 58.5% at 125 µg/mL, and 92.1 and 85.6% at 1000 µg/mL, using the clover and maize pollen grains extracts, respectively (Table 3). Moreover, the IC_50_ of the clover pollen grains extract was 22.18 µg/mL, while 54.85 µg/mL was recorded as the IC_50_ for the maize pollen grains extract. All these findings were compared to ascorbic acid as a standard drug which exhibited an IC_50_ value of 2.51 µg/mL. A previous study reported the antioxidant activity of maize pollen grains with an IC_50_ value of 425.4 µg/mL [28]. These differences in the antioxidant activity may depend on several factors, such as the plant origin, phenolic, and flavonoid constituents of pollen grains. 

In the present study, clover pollen grains possessed a high content of quercetin compared to maize pollen grains. The presence of these flavonoids showed a significant antioxidant activity of clover pollen grains compared to maize pollen grains. Leja et al. [29] reported that the phenolics, flavonoids, and pigments such as β-carotene were responsible for the action mechanism of antioxidants of pollen grains. Marghitaş et al. [30] mentioned that quercetin played a vital role in preventing the oxidative damage of cellular biomolecules, such as nucleic acids, and lipoprotein due to the reactive oxygen species (ROS). Strong antioxidant activity was attributed to maize pollen grains as mentioned by Bujang et al. [31]. Several investigations documented that pollen grains had significant antioxidant potential that was commonly influenced by the presence of phenolic and flavonoid constituents. However, a great difference of the antioxidant activity was observed between pollen grains obtained from various plant species and different geographical areas [32]. In a previous study, the antioxidant activity of the maize pollen grains extract had a lower IC_50_ value than the IC_50_ of clover [24]. According to Avşar et al. [2], the IC_50_ value of pollen grains of *Castanea sativa* via DPPH was 19.5 µg/mL. In the study conducted by Bakour et al. [19], the IC_50_ values of *P. granatum*, *Q. ilex*, and *C. erythraea* pollen grains extracts were 2, 8 and 200 µg/mL, respectively.

### 2.4. Anticancer Activity of Pollen Grains 

**Figure 4:**
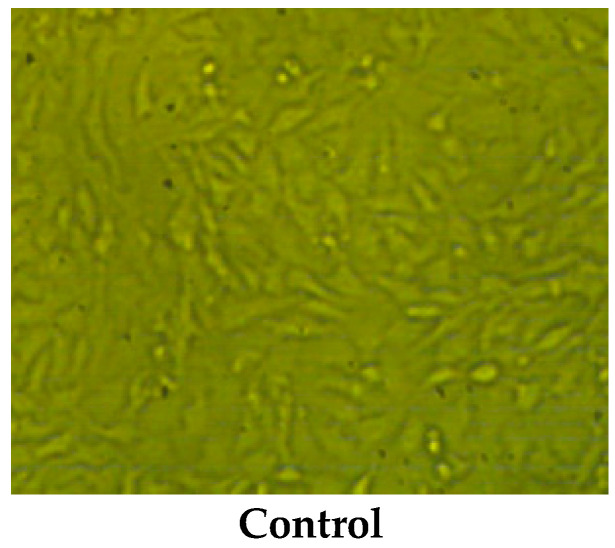

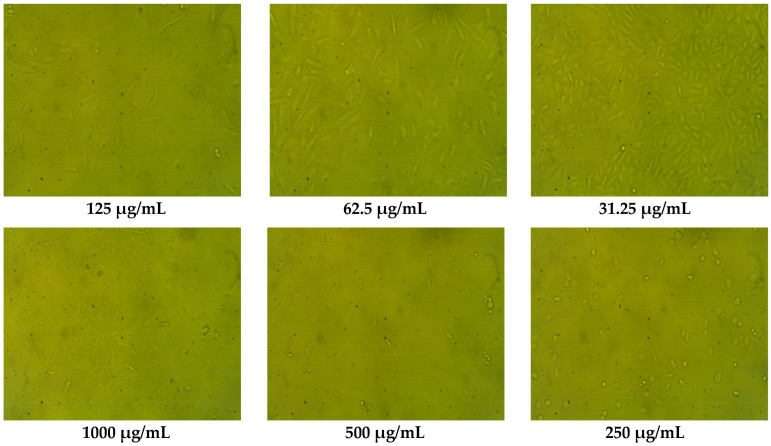
The morphological features of HCT-116 cells line (control) exposed to different concentrations of the maize pollen grains extract. As the concentration of maize pollen grains increased, cancerous cells (HCT-116) become tightly regulated within a cell suicide process.

**Figure 5:**
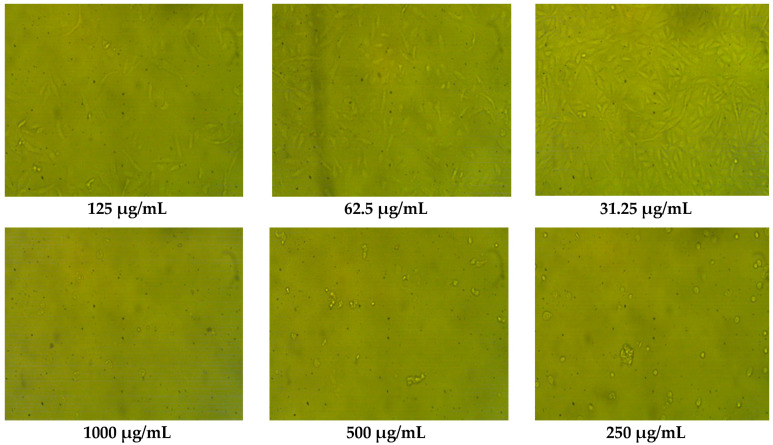
The morphological features of HCT-116 cells line exposed to different concentrations of the clover pollen grains extract. As the concentration of clover pollen grains increased, cancerous cells (HCT-116) become tightly regulated within a programed cell death process.

The anticancer potential of the two types of pollen grains was examined against human colon cancer cell line (HCT-116) (Table 4). It was clear that the maize pollen grains extract was more effective than clover pollen grains, particularly at concentrations up to 125 µg/mL. There were negligible differences in the cytotoxicity of the two pollen grains extracts at high concentrations (250–1000 µg/mL). A lower IC_50_ value (67.02 ± 1.37 µg/mL) of maize than the IC_50_ value (75.03 ± 1.02 µg/mL) of clover pollen grains was recorded compared with the IC_50_ value of the positive control Adriamycin (43.12 ± 1.25 µg/mL). Wang et al. [33] have reported the anticancer activity of *Rosa rugosa* pollen grains against HCT-116 and HT-29 cell lines, using MTT assay. The pollen grains of other origin, such as *Bauhinia variegate*, *Cassia biflora*, *C. siamea* and *C. glauca* exhibited anticancer activity [12]. The morphological study reported the presence of an alteration in the treated HCT-116 cells with the two pollen grains extracts at a concentration of more than 62.6 µg/mL increasing the damaged and dead cells (Figure 4 and Figure 5). Moreover, the detachment and rounding of HCT-116 cells were observed at 250–1000 µg/mL with a reduction in treated cells and cytoplasm mass. These alterations in the exposed cells to the maize and clover pollen grains extracts have documented some markers of antitumor activity against HCT-116 cells.

On the other hand, untreated HCT-116 cells remained in their identical spindle and angular form. Some detected compounds in the current pollen grains extract, such as naringenin, quercetin, kaempferol, and coumaric acid were reported as anticancer agents in vitro [34]. Maize pollen grains were tested against human prostate cancer by Ganash [28], where at 500 µg/mL, it exhibited 77.09% cytotoxicity with an IC_50_ value of 339.81 µg/mL, and the marker changes in the tested cells, such as shrunken and round form, and breakdown of cell DNA were reported. According to Elsayed et al. [18], beebread (mixture of plant pollen and honey fermented with lactic acid) had anticancer potential against liver hepatocellular, colorectal adenocarcinoma, and prostate adenocarcinoma cell lines of human with IC_50_ values of 386, 314 and 262 μg/ mL, respectively.

## 3. Materials and Methods

### 3.1. Pollen Grains Source and Extraction

**Figure 6:**
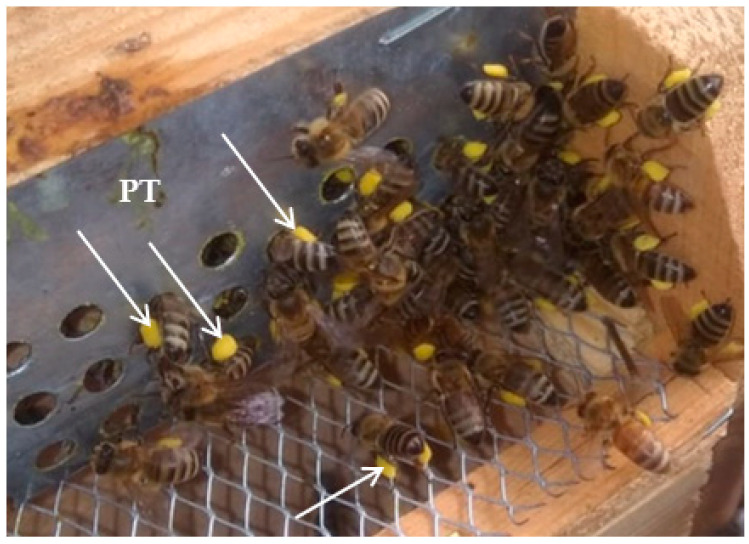
Pollen trap (PT) fitted to the entrance of a hive bee to collect pollen grains (white arrows).

In the current study, the pollen grains were harvested by bees via the fitting of a pollen trap at the hive entrance of the 10 bees for 3 days (Figure 6). The pollen grains of clover were dark yellow and collected during the season of clover (during the period of clover “May” flowering) from a farm that cultivated only clover, while maize pollen grains were yellow and collected during the season of maize from a farm that cultivated only maize (during the period of maize “July” flowering). Moreover, the farms cultivated only clover and maize. In addition to that, the farms were treated with herbicides to prevent other cultivars. Furthermore, the cultivated area was huge, ranging from 6 to 8 hectares. Therefore, further palynological analysis was not required. The harvested pollen grains were dehydrated at 40 °C for 12 h, using an oven. The extracts of the collected pollen grains were prepared by mixing 50 g into 500 mL of methanol. Then, via shaking in a water bath, they were shaken for 24 h, which was followed by filtration. Finally, the extracts of the pollen grains were concentrated to obtain a crude extract under vacuum.

### 3.2. Assessment of Phenolic and Flavonoid Constituents of Pollen Grains by HPLC

HPLC (Agilent 1260 series) was used to determine the phenolic and flavonoid contents in the pollen grain extracts. One Zorbax Eclipse Plus C8 column (4.6 mm × 250 mm i.e., 5 μm) was used for the separation process. The mobile phase (MP) consisted of acetonitrile and water (W) with 0.05% trifluoroacetic acid (A) flowing at a rate of 0.9 mL/min. The MP was automatically programmed in the following flowing order, using a linear gradient: 82% W for 0 min, 75% W for 1 min, 60% W for 11–18 min, and 82% W for 18–24 min. The ultraviolet (UV) detector was used to detect flavonoids at 330 nm and phenolic components at 280 nm. The tested sample solution was injected into a volume of 5 μL, while the column was kept at 40 °C. Utilizing standard molecules of flavonoids and phenolic acid as input data, the extract’s chemical composition was semi-quantitatively determined.

### 3.3. Tested Microorganisms and Cancer Cell Line

*Escherichia coli* ATCC 11293, *Staphylococcus aureus* ATCC 6538, *Bacillus Subtilis* (ATCC 6633), *Salmonella typhi* (ATCC 6539), *Candida albicans* ATCC 14053 and *Aspergillus niger* (Ain Shams University Hospitals and University Mycological Centre, Egypt) were investigated. The tested bacteria/fungi were activated using nutrient/Sabouraud Dextrose agar (SDA) media (Sigma–Aldrich, Steinheim, Germany). Regarding the cancer cell line, the human colon cancer cell line (HCT-116) (Organism: Homo sapiens, human; tissue: colon; cell type: epithelial; disease: colorectal carcinoma) was purchased from the American Type Culture Collection (ATCC, Rockville, MD, USA).

### 3.4. Antimicrobial Activity of Pollen Grains Extract

The well diffusion technique was used to examine the antimicrobial activity of the extracts of pollen grains. For antibacterial activity, 20 mL of medium growth (Mueller–Hinton agar) (Modern Lab Co., Indore, Madhya Pradesh, India) was poured onto plastic petri plates. A bacterial inoculum, containing 0.5 McFarland 2 × 10^8^ CFU/mL was added to each Petri plate containing Mueller–Hinton agar, while Sabouraud agar medium was used for fungi. Through sterile cork borer, 6 mm diameter wells were cut from the medium, which was followed by filling them with 20 μL of the methanolic extract of pollen grains. Solvent served as a negative control, but antibiotic was used as a positive control. The bacteria were incubated at 37 °C for 24 h, while fungi were incubated for at 30 °C 3 days. The zones of inhibition were measured in millimeters [35,36].

### 3.5. Minimal Bactericidal Concentration (MBC) and Minimum Inhibitory Concentration (MIC) of Pollen Grains Extract

Different dilutions of both extracts of pollen grains were prepared. From each dilution, 10 μL was added to 170 μL of Mueller–Hinton broth into 96 wells of a microplate amended with 20 μL of bacterial inoculum, containing 5 × 10^5^ CFU/mL based on the approach of NCCLS standards [35]. The microtiter plates were then incubated at 37 °C for 24 h. The developed turbidity indicates the growth of the tested microorganism, and the MIC is the lowest concentration of the tested extract where no growth is visually detected. The MBC is detected, where the dilution demonstrates the MIC and at least two of the highly concentrated compound dilutions are plated and counted to estimate the viable CFU/mL of the tested microorganisms. The MBC is the lowest dose that determines a pre-detected reduction (99.9%) in CFU/mL when compared to the MIC dilution.

### 3.6. Ultrastructure of E. coli

A JEOL-JEM 1010 transmission electron microscope (TEM) (JEOL JEM 1200-, Tokyo, Japan) was used to examine *E. coli* cells exposed to the pollen grains extract, where the treated *E. coli* cells were fixed in 3% glutaraldehyde and rinsed in phosphate buffer. Then, via a solution of potassium permanganate, the cells were post-fixed at 25 °C for 5 min. The treated cells were gradually dehydrated by ethanol (10% to 90% ethanol) at 25 °C. At the end, using the absolute ethanol, the cells were dehydrated for 30 min. The treated *E. coli* cells were infiltrated via epoxy resin and acetone until forming a pure resin. The ultrathin segments of treated cells were loaded on the grids of copper, which was followed by staining them twice with uranyl acetate and lead citrate. At 70 kV, the stained sections were imagined by TEM [37].

### 3.7. Antioxidant Activity of Pollen Grains Extract

Using the DPPH test, free radical scavenging activity was evaluated in accordance with the technique outlined by Al-Rajhi et al. [38]. Briefly, a PerkinElmer Lambda 40 UV/Vis spectrophotometer was used to measure the absorbance at 517 nm after adding 75 μL of various dilutions of the extracts of each pollen to an 825 μL solution of DPPH (63.4 μM, prepared in ethyl alcohol). Ascorbic acid was used as a positive control. The reaction was carried out in the dark for an hour. Using the following equation, the proportion of the DPPH radical scavenging inhibition was calculated:

$$Inhibition\left(\%\right)=\frac{Absorbanceatcontrol-Absorbanceattreatement}{Absorbanceatcontrol}\times 100$$

The graph of the curve representing the percentage of DPPH inhibition as a function of the pollen grains extract concentration was used to estimate the concentration of each extract needed to scavenge 50% of DPPH (IC_50_). The values for the IC_50_ were given in μg/mL.

### 3.8. Anticancer Activity of Pollen Grains Extract and Morphological Characteristics of Cancer Cells

The 3-(4,5-dimethyl-2-thiazolyl)-2,5-diphenyl-2H-tetrazolium bromide (MTT) test was used to determine the cytotoxicity activity of the pollen grains extract against the HCT-116 cell line. Prior to the MTT assay, cells (5 × 10^4^ cells/well) were dispersed into a 96-well sterile microplate and treated for 48 h at 37 °C with a series of varied doses of the pollen grains extract (31.25–1000 μg/mL) in DMSO, and Adriamycin was used as a positive control as well. Following incubation, the media were carefully withdrawn, each well was fortified with 40 µL of MTT (2.5 mg/mL), and incubation was again performed for 4 h at 37 °C at 5% CO_2_. Then, 200 µL of DMSO was added to dissolve the purple formazan dye crystals. A SpectraMax^®^ Paradigm^®^ Multi-Mode microplate reader was used to measure the absorbance at 560 nm and subtract the background at 620 nm. Optical density was directly linked with cell mass. In comparison with the untreated control cells, the mean percentage of viable cells was used to express the relative cell viability. Through a phase contrast microscope, the morphological characteristics of HCT-116 cell line cells were examined after 24 h of treatment [39,40].

### 3.9. Statistical Analysis 

Each value is the mean of 3 replicates ± standard error of means. An Honestly Significant Difference (HSD) at *p* ≤ 0.05 by post hoc Tukey’s test was applied. IC_50_ was detected by probit analysis, utilizing SPSS 29 software program (SPSS Inc., Chicago, IL, USA).

## 4. Conclusions

The current findings concluded that the tested extracts of pollen grains (maize and clover) held great potential for therapeutic applications due to the observed potent antimicrobial, antioxidant, and anticancer activities. Clover pollen grains exhibited stronger antimicrobial and antioxidant activities, while lower toxicity toward cancer cells (HCT-116) was monitored when compared to maize pollen grains. Furthermore, the morphological effects of the tested extracts (maize and clover pollen grains) against certain bacterial and cancer cells were represented by the ultrastructure changes in *E. coli* and HCT-116 cells, which confirmed the antibacterial and antitumor activities. Moreover, pollen grains represent a safe substitute in nutritional and pharmacological fields. Thus, maize and clover pollen grains and their derived phenolic and flavonoid compounds can serve as a future food additive for human consumption as well as a source of bioactive and functional constituents in pharmaceutical and nutraceutical industries. Nevertheless, the underlying mechanism of the identified constituents in the pollen grains extracts should be further in vitro examined against a wider range of cancer and bacterial cells.

## Figures and Tables

**Table 1:** The phenolic and flavonoid compounds detected in maize and clover pollen grains.

Compound	Pollen Grains Extract of Maize				Pollen Grains Extract of Clover			
	Retention Time	Area	Area (%)	Conc. (µg/mL)	Retention Time	Area	Area (%)	Conc. (µg/mL)
Gallic acid	3.544	22.87	2.0647	100.21	3.542	22.81	0.7373	99.97
Chlorogenic acid	4.300	15.59	1.4080	107.04	4.301	12.87	0.4159	88.33
Catechin	4.597	3.56	0.3218	41.14	4.714	4.03	0.1304	46.58
Methyl gallate	5.580	404.47	36.5194	1056.57	5.589	38.86	1.2556	101.50
Caffeic acid	6.087	17.13	1.5468	70.61	6.094	112.76	3.6439	464.73
Syringic acid	6.440	0.00	0.00	0.00	6.440	0.00	0.00	0.00
Pyro catechol	6.572	157.19	14.1924	1145.56	6.568	18.73	0.6052	136.48
Rutin	6.917	8.44	0.7624	68.41	6.925	0.00	0.00	0.00
Ellagic acid	7.531	1.45	0.1311	6.26	7.100	8.83	0.2854	38.09
Coumaric acid	8.299	94.09	8.4950	175.21	8.299	7.67	0.2479	14.29
Vanillin	8.942	26.10	2.3567	49.82	8.956	2.32	0.0748	4.42
Ferulic acid	9.802	1.92	0.1734	5.88	9.960	17.63	0.5698	53.98
Naringenin	10.590	12.37	1.1170	59.60	10.580	0.84	0.0273	4.07
Rosmarinic acid	11.723	1.75	0.1583	9.69	12.181	23.82	0.7697	131.66
Daidzein	16.203	2.60	0.2348	7.65	16.025	3.66	0.1183	10.77
Quercetin	17.276	8.63	0.7796	53.90	17.272	62.97	2.0347	393.05
Cinnamic acid	19.541	15.42	1.3923	14.38	19.184	19.26	0.6224	17.97
Kaempferol	20.559	6.59	0.5950	21.84	20.814	117.93	3.8108	390.93
Hesperetin	21.181	5.39	5.38755	13.98	21.181	6.16	0.1990	15.97

**Table 2:** Efficacy of maize and clover pollen grains against tested microorganisms with MIC, MBC, and MBC/MIC index detection.

Tested Microorganisms	Mean Inhibition Zone (mm)				*p* Value	MIC (µg/mL)		*p* Value	MBC (µg/mL)		*p* Value	MBC/MIC Index		*p* Value
	Maize	Clover	+ve C *	-ve C *		Maize	Clover		Maize	Clover		Maize	Clover	
*Bacillus subtilis*	42 ± 0.4 a	39 ± 0.3 b	33 ± 0.1 c	0.0	0.000	3.9 ± 0.33 a	3.9 ± 0.25 a	1.00	7.9 ± 0.35 a	3.9 ± 0.2 b	0.00	2.02 a	1.0 b	0.00
*Staphylococcus* *aureus*	19 ± 0.3 b	22 ± 0.2 a	16 ± 0.2 c	0.0	0.001	125 ± 4.0 a	62.5 ± 1.00 b	0.00	125 ± 1.53 a	125 ± 3 a	1.00	1.0 b	2.5 a	0.00
*Escherichia coli*	15 ± 0.4 b	18 ± 0.1 a	18 ± 0.3 a	0.0	0.016	250 ± 3.0 a	62.5 ± 0.07 b	0.00	500 ± 3.0 a	125 ± 4 b	0.000	2.0 a	2.5 a	0.288
*Salmonella typhi*	27 ± 0.1 ab	29 ± 0.3 a	25 ± 0.1 b	0.0	0.125	31.25 ± 1.33 a	31.25 ± 0.5 a	1.00	31.25 ± 1.53 b	62.5 ± 1 a	0.00	1.0 b	2.0 a	0.00
*Candida albicans*	22 ± 0.4 b	42 ± 0.4 a	21 ± 0.3 b	0.0	0.000	15.62 ± 0.35 a	1.97 ± 0.04 b	0.00	15.62 ± 1.32 a	3.97 ± 0.2 b	0.002	1.0 b	2.01 a	0.00
*Aspergillus niger*	NA	NA	20 ± 0.4	0.0	0.000	-	-		-	-		-	-	

* +ve C, positive control (Gentamycin/Nystatin); -ve C, negative control (solvent of extraction used). Each value is mean of 3 replicates ± standard error of means. Honestly Significant Difference (HSD) at *p* ≤ 0.05 by post hoc Tukey’s test. The comparison refers to the means of the same row. The same letters mean no significance, while different letters mean significant difference.

**Table 3:** Antioxidant activity of maize pollen grains extract, clover pollen grains extract and ascorbic acid.

Concentration (µg/mL)	DPPH Scavenging (%)			*p* Value
	Maize	Clover	Ascorbic Acid	
1000	85.6 ± 0.05 ^c^	92.1 ± 0.01 ^b^	99.2 ± 0.02 ^a^	0.000
500	74.1 ± 0.17 ^c^	84.4 ± 0.02 ^b^	96.1 ± 0.031 ^a^	0.002
250	66.2 ± 0.24 ^c^	76.2 ± 0.04 ^b^	94.6 ± 0.14 ^a^	0.000
125	58.5 ± 0.16 ^c^	68.1 ± 003 ^b^	91.8 ± 0.25 ^a^	0.000
62.50	51.3 ± 0.32 ^c^	61.4 ± 0.05 ^b^	84.3 ± 0.41 ^a^	0.001
31.25	43.3 ± 0.21 ^c^	53.9 ± 0.15 ^b^	76.2 ± 0.08 ^a^	0.005
15.63	35.8 ± 0.45 ^c^	46.3 ± 0.24 ^b^	67.7 ± 0.24 ^a^	0004
7.81	28.2 ± 0.21 ^c^	38.4 ± 0.18 ^b^	60.4 ± 0.21 ^a^	0.003
3.90	20.4 ± 0.32 ^c^	30.7 ± 0.06 ^b^	52.2 ± 0.41 ^a^	0.005
1.95	12.1 ± 0.15 ^c^	24.1 ± 0.09 ^b^	43.6 ± 0.14 ^a^	0.000
0.0	0.0	0.0	0.0	
IC_50_	54.85 ± 0.21 ^a^ µg/mL	22.18 ± 0.21 ^b^ µg/mL	2.51 ± 0.24 ^c^ µg/mL	0.000

Each value is mean of 3 replicates ± standard error of means. Honestly Significant Difference (HSD) at *p* ≤ 0.05 by post hoc Tukey’s test. The comparison referred to the means of the same row. The same letters mean no significance, while different letters mean significant difference.

**Table 4:** Anticancer activity of maize and clover pollen grains extract against HCT-116 cells line.

Concentration (µg/mL)	Cytotoxicity (%)		*p* Value
	Maize	Clover	
1000	97.57 ± 0.24 ^a^	97.53 ± 0.21 ^a^	1.00
500	97.61 ± 0.16 ^a^	96.53 ± 0.02 ^a^	0.288
250	94.92 ± 0.08 ^a^	95.05 ± 0.15 ^a^	0.168
125	81.99 ± 0.07 ^a^	76.82 ± 0.65 ^b^	0.038
62.50	60.81 ± 0.14 ^a^	55.38 ± 0.57 ^b^	0.028
31.25	19.84 ± 0.35 ^a^	13.06 ± 0.38 ^b^	0.021
0.0	0.0	0.0	-
IC_50_ (µg/mL)	67.02 ± 1.37 ^b^	75.03 ± 1.02 ^a^	0.004

Each value is mean of 3 replicates ± standard error of means. Honestly Significant Difference (HSD) at *p* ≤ 0.05 by post hoc Tukey’s test. The comparison referred to the means of the same row. The same letters mean no significance, while different letters mean significant difference.

## Data Availability

Data are contained within the article and Supplementary Materials.

## Supplementary Materials

The following supporting information can be downloaded at https://www.mdpi.com/article/10.3390/ph16121731/s1, Figure S1: Chromatogram of detected phenolic and flavonoid compounds in maize via HPLC; Figure S2: Chromatogram of detected phenolic and flavonoid compounds in clover via HPLC.
