# Supplementary material for: Antimicrobial, Anticancer, and Antioxidant Activities of Maize and Clover Pollen Grains Extracts: A Comparative Study with Phytochemical Characterizations
Source: Pharmaceuticals (Basel). 2023 Dec 15;16(12):1731. doi: 10.3390/ph16121731 (PMC10747423; doi:10.3390/ph16121731)
Supplement: Supplementary file 1 [file pharmaceuticals-16-01731-s001.zip › pharmaceuticals-2748995-supplementary.pdf]

pharmaceuticals

Article Supplementary Materials

Antimicrobial, Anticancer, and Antioxidant Activities of Maize and Clover Pollen Grains Extracts: A Comparative Study with Phytochemical Characterizations

Heba Barnawi [1], Husam Qanash [1,2], Abdu Aldarhami [3], Ghaida Alsaif [1], Bandar Alharbi [1], Majed N. Almashjary [4,5], Alhomidi Almotiri [6], Abdulrahman S. Bazaid [1,2,*]

[1] Department of Medical Laboratory Science, College of Applied Medical Sciences, University of Ha'il, Hail 55476, Saudi Arabia; h.barnawi@uoh.edu.sa (H.B.); h.qanash@uoh.edu.sa (H.Q.); g.alsaif@uoh.edu.sa (G.A.); b.alharbi@uoh.edu.sa (B.A.); ar.bazaid@uoh.edu.sa (A.S.B.)
[2] Medical and Diagnostic Research Center, University of Ha'il, Hail 55473, Saudi Arabia
[3] Department of Medical Microbiology, Qunfudah Faculty of Medicine, Umm Al-Qura University, Al-Qunfudah 21961, Saudi Arabia; ahdarhami@uqu.edu.sa (A.A.)
[4] Department of Medical Laboratory Sciences, Faculty of Applied Medical Sciences, King Abdulaziz University, Jeddah 22254, Saudi Arabia; malmashjary@kau.edu.sa (M.N.A.)
[5] Hematology Research Unit, King Fahd Medical Research Center, King Abdulaziz University, Jeddah 22254, Saudi Arabia
[6] Department of Clinical Laboratory Sciences, College of Applied Medical Sciences-Dawadmi, Shaqra University, Dawadmi 17464, Saudi Arabia; hsalmutiri@su.edu.sa (A.A.)
* Correspondence: ar.bazaid@uoh.edu.sa (A.S.B.)

This document includes:

- Supplementary figures:

Figure S1: Chromatogram of detected phenolic and flavonoid compounds in maize via HPLC.

Figure S2: Chromatogram of detected phenolic and flavonoid compounds in clover via HPLC.

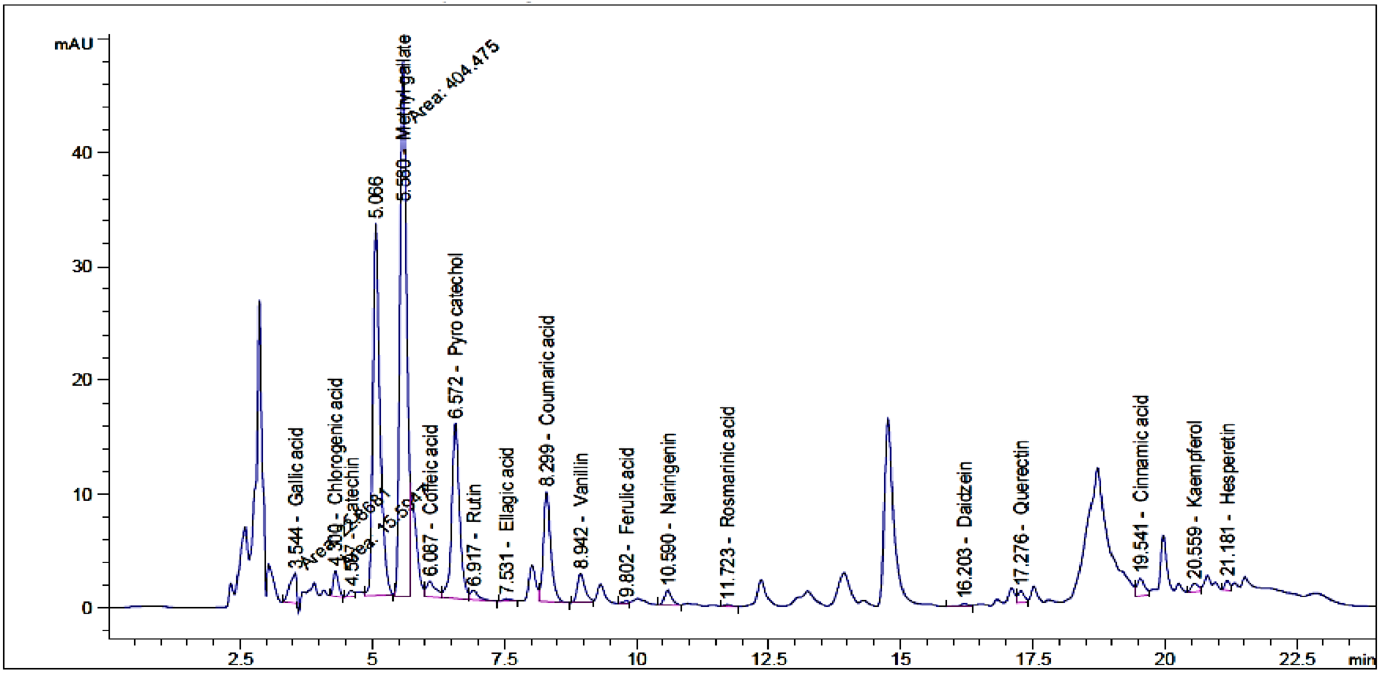

Figure S1. Chromatogram of detected phenolic and flavonoid compounds in maize via HPLC.

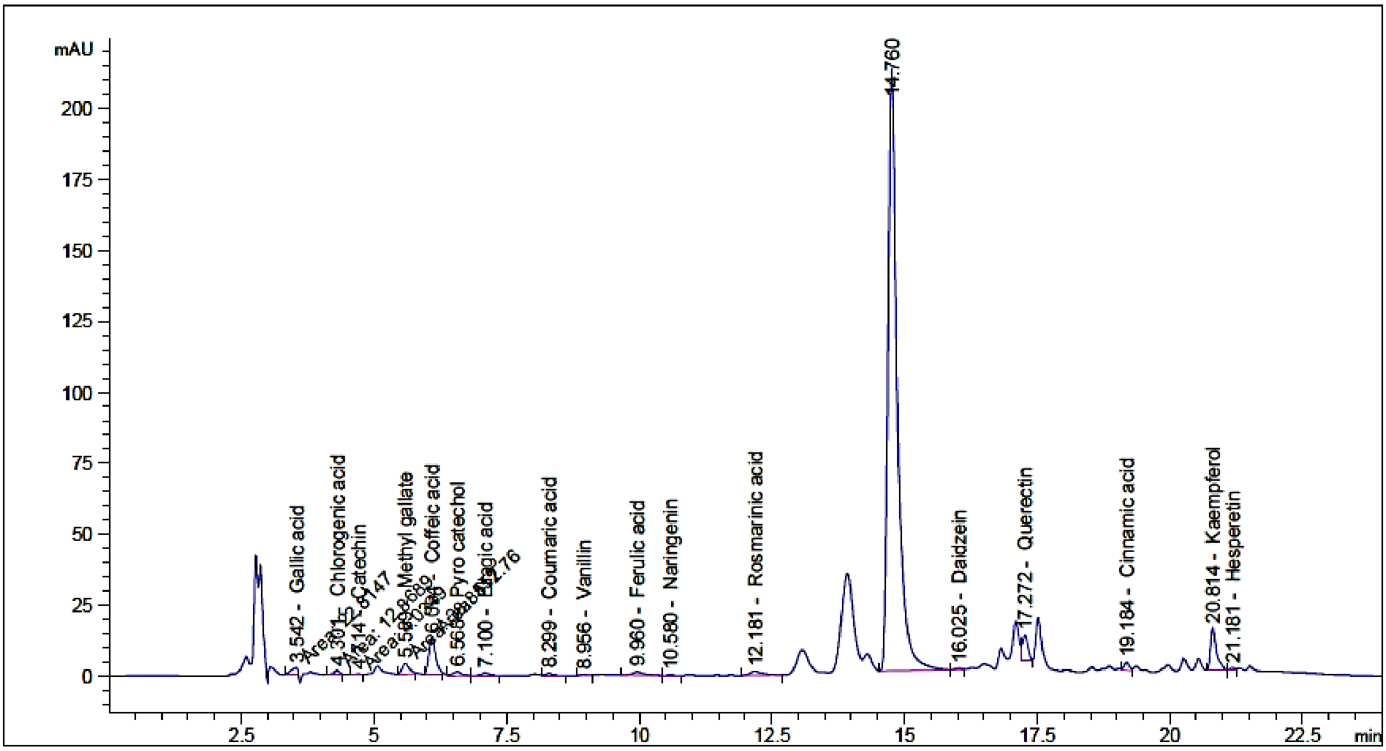

Figure S2. Chromatogram of detected phenolic and flavonoid compounds in clover via HPLC.
